# Ginsenoside Rb1 Reduces Isoproterenol-Induced Cardiomyocytes Apoptosis *In Vitro* and *In Vivo*

**DOI:** 10.1155/2013/454389

**Published:** 2013-11-18

**Authors:** Xiu-feng Wang, Xin-jun Liu, Qian-mei Zhou, Jia Du, Tian-ling Zhang, Yi-yu Lu, Shi-bing Su

**Affiliations:** ^1^Fujian Academy of Traditional Chinese Medicine, Fuzhou, Fujian 350003, China; ^2^Obstetrics & Gynecology Hospital of Fudan University, Shanghai 200090, China; ^3^Research Center for Traditional Chinese Medicine Complexity System, Shanghai University of Traditional Chinese Medicine, 1200 Cailun Road, Pudong, Shanghai 201203, China

## Abstract

Cardiomyocytes apoptosis can lead to heart failure. Conventional and alternative drugs, such as Chinese herbal remedies, have been developed to target cardiomyoblast cells apoptosis. In this study, we investigated the effects of ginsenoside Rb1 (Rb1), an active compound, which is isolated from Notoginseng and Ginseng on isoproterenol-(ISO-) induced apoptosis in rat cardiomyocytes and its mechanism *in vivo* and *in vitro*. Rb1 reduced the ISO-induced apoptosis in rat cardiomyocytes and H9c2 cells. The effect of Rb1 was significantly suppressed by H89 (inhibitor for PKA), but not by C-1 (inhibitor for PKC). Based on in-cell blot analysis, the ISO-induced PKA and PKC expressions were decreased by Rb1, which was inhibited by H89, but not by C-1. The expressions of caspase-3 and caspase-9 were decreased after treatment with both ISO and Rb1, but with no change for caspase-8. Our results indicated that Rb1 reducing ISO-induced rat cardiomyocytes apoptosis may be involved in PKA and caspase-9 pathways.

## 1. Introduction

Cardiomyocytes apoptosis is a potential mechanism in the heart disease. It has been known that stimulation of the beta-adrenergic agonists causes hypertrophy and apoptosis in cardiomyocytes [1, 2], which leads to further deterioration of cardiac function [3] and so far to an intensification of heart failure [4, 5]. Although adult cardiomyocytes are terminally differentiated and have lost their ability to divide, cardiomyocytes apoptosis may play an important role in heart disease. It can be considered a new approach to reduce or prevent inappropriate cardiac cell death in finding effective drugs as a therapeutic means of slowing down the loss of myocytes.

Recently, it was reported that there are some active compounds in Chinese herbal medicines which could inhibit cardiovascular disease-associated cell apoptosis or protect cardiomyocytes death. For example, silibinin efficiently protected beta-adrenergic agonist-induced rat neonatal cardiomyocytes injury [6, 7]. In H9c2 cardiomyocytes, reservation decreased apoptosis, ROS production, and intracellular calcium mobilization induced by treatment with As_2_O_3_ [8].

**Figure 1:**
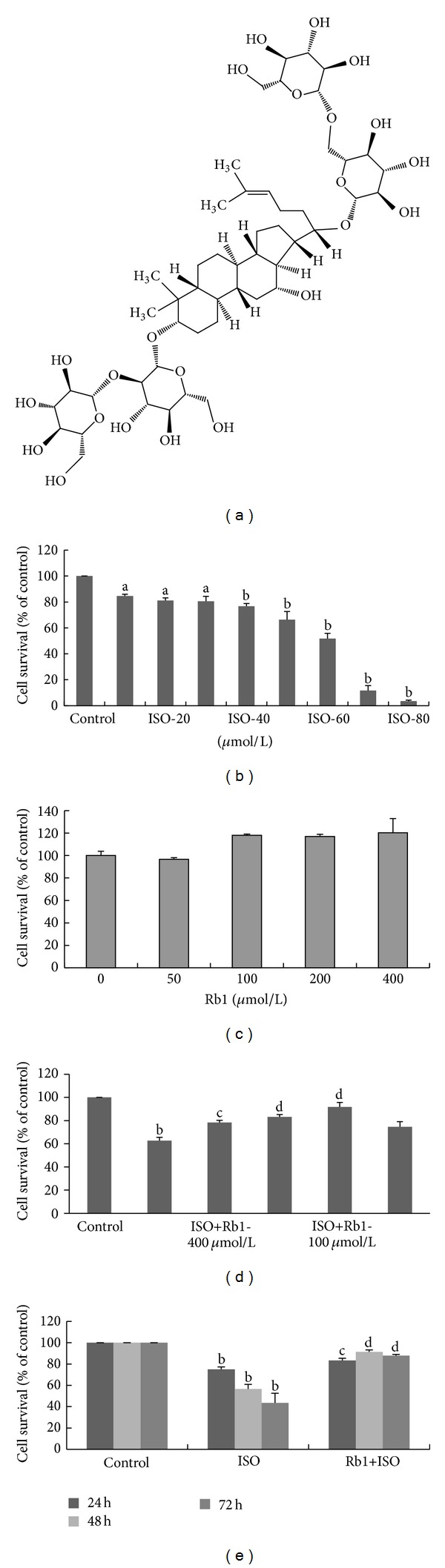
The effect of Rb1 on the survival of H9c2 cells. The cells were cultured in either presence or absence of ISO (60 *μ*mol/L). Data was expressed as percent of control and was the mean ± SD of three replicates. (a) The molecular structure of Rb1. (b) Cell survival rates on the different concentrations of ISO treatment. (c) Cell survival rates on the different concentration of Rb1 treatment. (d) The effects of Rb1 on the different concentrations of ISO treatment. (e) Cell survival rates on the Rb1 (100 *μ*mol/L) and/or ISO treatment for 24 h, 48 h, and 72 h. ^a^ *P* < 0.05, ^b^ *P* < 0.01 versus control, ^c^ *P* < 0.05, ^d^ *P* < 0.01 versus ISO.

Ginsenoside Rb1 (Rb1) (Figure 1(a)) is an active compound, which is isolated from Notoginseng and Ginseng in Chinese herbal medicine. It has been reported to attenuate atherosclerosis in rats by regulating the blood lipid profile and an anti-inflammatory action [9]. Moreover, Rb1 clearly alleviated cardiac dysfunction and remodeling in the cTnT^R141W^ transgenic mouse, attenuated cardiac hypertrophy, interstitial fibrosis, ultrastructural degeneration, and intercalated disc remodeling in dilated cardiomyopathy hearts [10], and promoted glucose-stimulated insulin secretion and survival in Min6 cells through PKA which augmented IRS2 expression to enhance insulin/IGF-1 signaling [11]. It is also resistant to anoikis and blocked Erk1/2 phosphorylation in the TKO MEFs [12], inhibited calcineurin signalling pathway in cardiomyocyte hypertrophy induced by prostaglandin F2alpha [13], and protected cardiomyocytes against CoCl_2_-induced apoptosis in neonatal rats by inhibiting mitochondria permeability transition pore opening [14]. However, whether Rb1 reduces cardiomyocytes apoptosis and what are the molecular mechanisms remain poorly understood.

In this study, we hypothesized that Rb1 is a novel agent for reducing isoproterenol-(ISO-) induced apoptosis. We aimed to examine the effects of Rb1 on ISO-induced cardiomyocytes apoptosis *in vivo *and* vitro* and determined the underlying apoptosis-related signaling mechanisms.

## 2. Materials and Methods

### 2.1. Reagents

Rb1 was obtained from the Standardization Center of Chinese Medicines Centre (Shanghai, China). The purity of Rb1 was measured by HPLC and was determined to be about 99%. Rb1 was dissolved in deionized water to make a stock solution. Caspase-3, caspase-8, caspase-9, GAPDH, and PKA antibodies were purchased from Cell Signaling Technology Inc. (Danvers, MA, USA). ISO, 3-(4,5-dimetrylthiazol-2-yl)-2,5-diphenyltetrazolium bromide (MTT), and Hoechst 33258 were purchased from Sigma Chemical Co. (St. Louis, MO, USA). 

### 2.2. Animals and Treatment Protocol

Sprague-Dawley rats, male, 210 ± 10 g, were provided by the Experimental Animal Center, Shanghai University of Traditional Chinese Medicine (Shanghai, China). They were fed in standard cages and maintained on a standard laboratory diet. The rats were treated by ISO as a myocytes apoptosis model [4, 15]. Control treatment group was injected with saline (1 mL/(kg·d), i.p, *n* = 10). The treatment groups were respectively treated by Rb1 (20 mg/(kg·d), i.p., *n* = 6) for 7 days, ISO was administered intraperitoneally with one-daily injections (5 mg/(kg·d)) for the last 3 days. After 7 days of the experimental regimen, the hearts wereexcised under anesthesia using sodium pentobarbital (50 mg/kg, i.p.). Then, left ventricle (LV) tissues were separated up, rinsed in iced sterile saline, placed in 10% buffered formalin, and processed for TUNEL staining.

### 2.3. Cell Line and Culture

H9c2 cells, a cardiomyoblast cell line derived from embryonic rat heart tissue, were obtained from the Shanghai Biological Sciences Institutes (Shanghai, China). The cells were maintained in DMEM (Gibco, Scotland, UK) supplemented with 10% FBS (Hyclone, Logan, UT, USA) and 100 U/mL penicillin/streptomycin in a 5% CO_2_ incubator at 37°C in a humidified atmosphere. 

### 2.4. Cell Viability Assay

Cell viability was assessed by MTT. Cells were seeded on 96-well plates at a density of 5 × 10^3^ cells per well. After 12 h, medium was changed to DMEM plus 5% fetal bovine serum with ISO (60 *μ*mol/L) or ISO (60 *μ*mol/L) + Rb1 (100 *μ*mol/L) with or without H89 or C-1 for 24, 48, and 72 h, respectively. Rb1 was added for 40 min prior to ISO treatment. Then, cells were incubated with MTT (1 mg/mL) for 4 h. The cells viability was assessed at 490 nm absorbance using a 96-well plate reader (Biotek, VT, USA). The viability was calculated as viability (%) = (*A*_490,sample_ − *A*_490,blank_)/(*A*_490,control_ − *A*_490,blank_) × 100.

### 2.5. Flow Cytometry Analysis

H9c2 cells were seeded in 60 mm dishes in DMEM plus 10% FBS. After 12 h, medium was changed to DMEM plus 5% FBS with ISO (60 *μ*mol/L) or ISO (60 *μ*mol/L) + Rb1 (100 *μ*mol/L). Cells were treated without or with H89 (5 *μ*mol/L) and without or with C-1 (100 nmol/L) for 48 h. Rb1 was added for 40 min prior to ISO treatment. Cells were collected after 48 h. The first stained with FITC-conjugated Annexin V for 30 min, and then stained with propidium iodide (PI) before 1 min and analyzed by FACScan (Beckman Coulter, FL, USA). The stainings were carried out using Annexin V/PI apoptosis kit (Beckman Coulter) according to the manufacture. Detection and quantification of apoptotic cells were obtained by flow cytometry analysis software (Cell Lab Quanta Analysis, Beckman Coulter).

### 2.6. Hoechst 33258 Staining

H9c2 cells (5 × 10^4^/well) were seeded in 6-well plates with cover slips and left overnight. When the cells anchored to the plates, ISO (60 *μ*mol/L) and/or Rb1 (100 *μ*mol/L) were added. Cells were treated without or with H89 (5 *μ*mol/L) for 24 h. Rb1 was added for 40 min prior to ISO treatment. After incubation for 24 h, the cells were fixed with 1 mL of 4% paraformaldehyde for 20 min. Then, the cells were incubated in 1 mL PBS containing 10 *μ*mol/L Hoechst 33258 at 37°C for 30 min and observed using fluorescence microscopy (Olympus, Tokyo, Japan) at ×400 magnification.

### 2.7. *In Situ* Labeling of DNA Fragments

DNA fragmentation in the myocytes of LV tissues was detected *in situ* by using terminal deoxyribonucleotide transferase-(TdT-) mediated dUTP nick-end labeling (TUNEL) kit (Kai-ji, Nanjing, Jiangsu, China). Briefly, after incubation with proteinase K (20 mg/mL), DNA fragments in the tissues sections were labeled with 2 nmol/L biotin-conjugated dUTP and 0.1 U/mL TdT at 37°C for 1 h. Nuclei exhibiting DNA fragmentation were visualized by incubation in 3,3-diamino benzidine (DAB). The sections were observed by light microscopy. The nuclei of apoptotic cells were stained dark brown. At the same magnification (×400), a minimum of 10 fields with myocytes cut in cross section from each LV tissues were examined to count TUNEL-positive cardiomyocytes. 

### 2.8. In-Cell Western Assay

The in-cell protein levels were determined by in-cell western assay as a previous report [16]. The cells (1 × 10^4^/well) were seeded on 96-well plate and incubated for 72 h. Then cells were incubated with vehicle, ISO (60 *μ*mol/L), ISO (60 *μ*mol/L) + Rb1 (100 *μ*mol/L), without or with H89 (5 *μ*mol/L) and without or with C-1 (100 nmol/L) for 24 h in DMEM plus 5% FBS. Rb1 was added for 40 min prior to ISO. Then the cells were immediately fixed with 4% formaldehyde for 20 min. After washing with 0.1% Triton, cells were blocked by 10% nonfat milk for 90 min. The cells were then incubated with diluted primary antibodies PKA and PKC (1 : 100), caspases 3, 8 and 9 (1 : 200), respectively. GAPDH was added to each well at the same time as control. After being treated at 4°C overnight, the cells were then incubated with corresponding second IRDyeTM700DX (red) or IRDyeTM800DX (green) fluorescence antibody for 2 h. The image was obtained by Odyssey Infrared Imaging System (Li-cor Biosciences, NE, USA). The protein levels were calculated as the ratio of the intensity of PKA, PKC, caspase-3, caspases-8 and caspases-9 to that of GAPDH. The experiments were carried out in triplicate and repeated three times.

### 2.9. Statistical Analysis

All data were presented as mean ± SD and were analyzed using SPSS 11.5 software. Comparisons among groups were made by an unpaired Student's *t*-test. A value of *P* < 0.05 was considered statistically significant.

## 3. Results

### 3.1. Rb1 Reduced ISO-Induced Cell Death in H9c2 Cells

According to previous reports [2, 7] that ISO could induce cell death and that it was carried by *β*-adrenergic receptor (*β*-AR) in H9c2 cells, in our study, the effect of Rb1 on the survival of H9c2 cells was evaluated by MTT assay. As expected, it was shown to have an ISO concentration-dependent decrease (Figure 1(b)). Rb1 alone at 50, 100, 200, and 400 *μ*mol/L increased cell growth (Figure 1(c)), and Rb1 increased cell survival under ISO (60 *μ*mol/L, a closed to IC50 concentration) for 48 h. The best effect was 100 *μ*mol/L (Figure 1(d)). It markedly counteracted ISO-induced cell death and restored survival up to 91.78%. Furthermore, the effects of Rb1 on the survival of H9c2 cells were evaluated for 24 h, 48 h, and 72 h, respectively. The survival rates were 83.32% for 24 h, 91.37% for 48 h, and 87.89% for 72 h (Figure 1(e)). These results suggested that Rb1 reduced ISO-induced H9c2 cell death. 

### 3.2. Rb1 Reduced ISO-Induced H9c2 Cell Death via PKA Pathway

**Figure 2:**
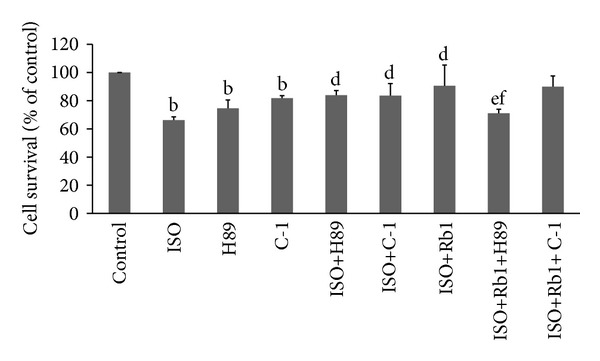
Cell survivals by the treatment of PKA and PKC inhibitors. The survival of H9c2 cells was evaluated by MTT assay after the treatments of Rb1 and/or ISO alone or combined with H89 or C-1. The tripleexperiment of results was expressed as mean ± SD, ^b^ *P* < 0.01, versus control; ^d^ *P* < 0.01, versus ISO; ^e^ *P* < 0.01, versus Rb1+ISO; ^f^ *P* < 0.01, versus Rb1+ISO+C-1.

It has been reported that PKA or PKC pathway plays an important role in cardiomyocytes cells survival [7, 17, 18]. In this study, H89, a PKA inhibitor, and C-1, a PKC inhibitor were used to investigate the relationship between Rb1 effect and PKA or PKC pathway. As shown in Figure 2, the ISO-induced H9c2 cell death was significantly decreased not only by H89 but also by C-1, compared to ISO-treated cells (*P* < 0.01). However, there was no significant difference between ISO+H89-treated cells and H89-treated cells (*P* > 0.05), as well as ISO+C-1-treated cells and C-1-treated cells (*P* > 0.05). When Rb1 was present, the ISO-induced H9c2 cell death was significantly decreased, compared to ISO-treated cells (*P* < 0.01). Moreover, the ISO+Rb1-treated H9c2 cell death was significantly increased by H89 (*P* < 0.01), and not by C-1 (*P* > 0.05), compared to ISO+Rb1-treated H9c2 cells. Furthermore, there was significant difference between ISO+Rb1+H89-treated cells and ISO+Rb1+C-1-treated cells (*P* < 0.01). These findings indicated that the Rb1 reduced ISO-induced cell death which may be mainly through the PKA pathway, rather than PKC signaling pathway.

### 3.3. Rb1 Reduced ISO-Induced Apoptosis in H9c2 Cells by Flow Cytometry Assay

**Figure 3:**
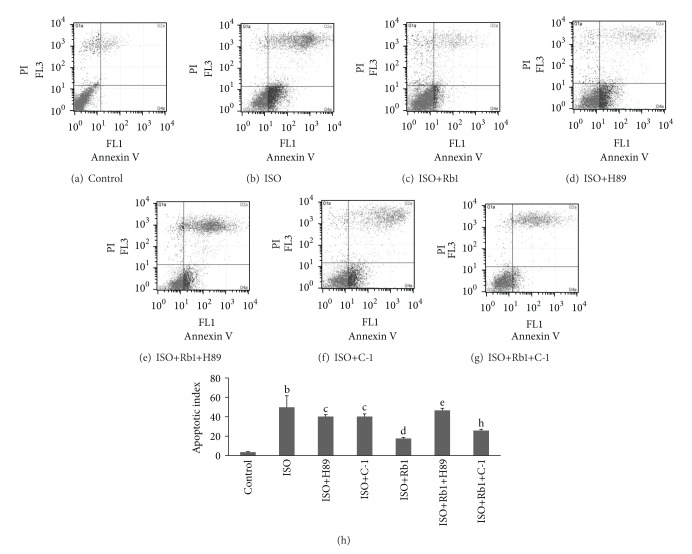
Apoptotic rates in H9c2 cells by flow cytometry assays. H9c2 cells were incubated with Annexin V-FITC and PI and analyzed in flow cytometry. (a) Control. (b)–(g) represent each treatment group. *X*-axis indicated the numbers of Annexin V-FITC stained cells as FL-1. The *Y*-axis indicated the numbers of PI strained cells as FL-3. The percentages indicated on the graph are the percent of double positive PI and annexin V-stained cells. (h) Statistical graph of annexin V-FITC/PI staining. The tripleexperiment of results was expressed as mean ± SD, ^b^ *P* < 0.01 versus control; ^d^ *P* < 0.01, ^c^ *P* < 0.05 versus ISO; ^e^ *P* < 0.01 versus Rb1+ISO; ^h^ *P* < 0.01 versus ISO+Rb1+C-1.

To further reveal the effect of Rb1 on the apoptosis event, we next examined apoptosis on Rb1-treated H9c2 cells in response to ISO-treated by evaluating the percentage of PI and annexin-V stained cells. As shown in Figure 3, the percentages of the annexin V/PI-double positive-stained cells in ISO-treated group dramatically increased compared to control group (49.8 ± 11.90% versus 3.54 ± 0.60%, *P* < 0.01) (Figures 3(a) and 3(b)). H89 and C-1 significantly decreased positive-stained cells by ISO-treated compared to only ISO-treated cells (40.21 ± 1.99% versus 49.8 ± 11.90%; 40.23 ± 2.69% versus 49.8 ± 11.90%, *P* < 0.01) (Figures 3(d) and 3(f)), Rb1-treated positive-stained cells were remarkably lower than ISO-treated cells (17.65 ± 0.99% versus 49.8 ± 11.90%, *P* < 0.01) (Figure 3(c)). However, the ISO+Rb1-treated positive-stained cells were significantly increased by H89 (46.53 ± 2.25% versus 17.65 ± 0.99%, *P* < 0.01) (Figure 3(e)), and not by C-1 (25.80 ± 1.31% versus 17.65 ± 0.99%, *P* > 0.05) (Figure 3(g)), compared to ISO+Rb1-treated cells. Furthermore, there was significant difference between ISO+Rb1+C-1-treated cells and ISO+C-1-treated cells (25.80 ± 1.31% versus 40.23 ± 2.69%, *P* < 0.01). These results suggested that Rb1 reduced ISO-induced H9c2 cells apoptosis via PKA pathway. 

### 3.4. Rb1 Reduced H9c2 Cell Apoptosis by the Morphological Observation

**Figure 4:**
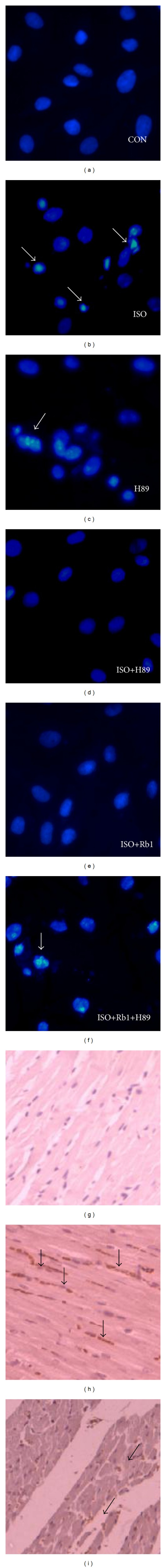
Morphologic changes in H9c2 cells and in LV tissues of rats. H9c2 cells were stained with Hoechst 33258 and the sections of LV tissue were stained with TUNEL. They were visualized under fluorescence or light microscope (magnifications: ×400). (a) Control cells; (b) ISO-treated H9c2 cells; (c) H89-treated H9c2 cells; (d) ISO+H89-treated H9c2 cells; (e) ISO+Rb1-treated H9c2 cells; (f) ISO+Rb1+H89-treated H9c2 cells; (g) control LV tissues in rat; (h) ISO-treated LV tissues in rat; (i) ISO+Rb1-treated LV tissues in rat. Arrows indicate apoptotic cells.

To further verify the effects of Rb1 on ISO-induced apoptosis in H9c2 cells, Hoechst 33258 staining was performed. Without ISO treatment (control group), the nuclei were stained a less bright blue and the color was homogeneous (Figure 4(a)). However, when cells were treated with ISO (60 *μ*mol/L) for 24 h, the staining showed the morphological changes in the nuclear chromatin and showed that the blue emission light in apoptotic cells was much brighter than that in the control cells. The condensed chromatin and fragmented nuclei were found in many treated cells, as the classic characteristics of apoptotic cells (Figure 4(b)). In the Rb1 pretreated group, the morphological changes were not observed (Figure 4(e)). When cells were treated with H89 alone (inhibitor for PKA), the condensed chromatin and fragmented nuclei were increased (Figure 4(c)). When H89 was further added, Rb1-pretreated morphological changes were not found (Figure 4(f)). These results suggested that Rb1 reduced apoptotic cells stimulated by ISO, which can be inhibited by H89.

### 3.5. Rb1 Reduced TUNEL-Positive Cardiomyocytes in Rat

**Figure 5:**
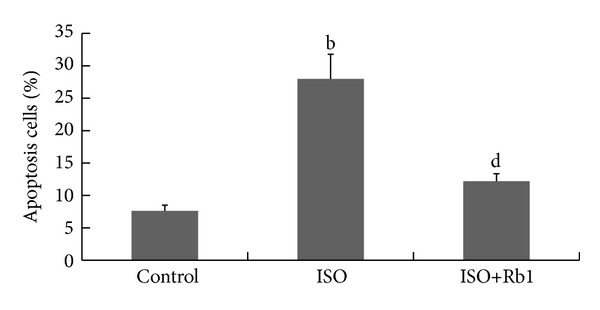
The apoptotic index of cardiomyocytes in rat. By TUNEL assay, 10 fields with myocytes cut in cross section from each LV tissue were examined to count TUNEL-positive cardiomyocytes. The apoptotic index was measured by TUNEL-positive cardiomyocytes to the total of cardiomyocytes, and results are expressed as means ± SD, ^b^ *P* < 0.01 versus control; ^d^ *P* < 0.01 versus ISO.

To evaluate the cardiomyocytes apoptosis* in vivo*, the sections of LV tissue were detected by TUNEL assay. It revealed only small numbers of TUNEL-positive cells in the control group (Figure 4(g)). The cardiomyocytes in the normal part were of regular shape, and counterstaining was blue. There was many TUNEL positive cardiomyocytes (brown) in ISO-treated group (Figure 4(h)). However, there was only a few TUNEL positive cardiomyocytes in Rb1-treated group (Figure 4(i)). As shown in Figure 5, the apoptotic index (the ratio of apoptotic myocytes to the total of cardiomyocytes) was significantly higher in ISO-treated group than in control group (*P* < 0.01) and was significantly lower in Rb1-treated group than in ISO-treated group (*P* < 0.01). These results suggested that Rb1 reduced ISO-induced cardiomyocytes apoptosis in rats. 

### 3.6. Rb1 Reduced ISO-Induced the Expressions of PKA Further, PKC, Caspase-3, and Caspase-9 in H9c2 Cells

**Figure 6:**
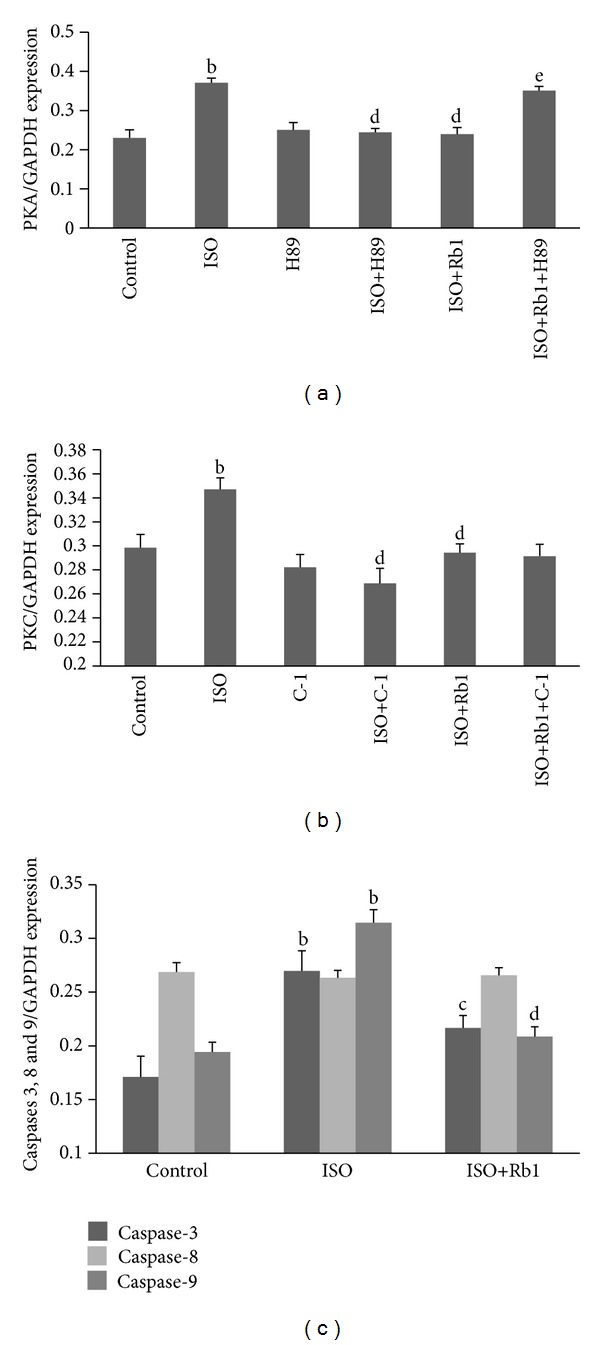
The expressions of PKA, PKC, caspase-3, caspase-8, and caspase-9. The protein expressions were assessed by in-cell Western Blot assay and Odyssey Infrared Imaging System after the treatments of Rb1 and/or ISO alone or combined H89 or C-1. The protein expressions were normalized to GAPDH and reported as percent of basal. (a) PKA expression; (b) PKC expression; (c) caspase-3, caspase-8 and caspase-9 expressions. The pictures were from the representative one of three individual experiments. ^b^ *P* < 0.01 versus control; ^d^ *P* < 0.01, ^c^ *P* < 0.05 versus ISO; ^e^ *P* < 0.05 versus Rb1+ISO.

In order to clarify the effects of Rb1 on ISO-induced expressions of PKA, PKC, caspase-8, and caspase-9 in H9c2 cells, an in-cell western blot assay was carried out. As shown in Figure 6, ISO increased PKA expression compared to control (*P* < 0.01). H89 and C-1 significantly decreased ISO-induced PKA expression, compared to ISO-treated cells (*P* < 0.01). Rb1 prevented the expressions of PKA and PKC which was increased by ISO, compared to ISO-treated cells (*P* < 0.01). Preincubation with H89 significantly decreased the beneficial effects of Rb1 on PKA expression, compared to ISO+Rb1-treated cells (*P* < 0.01) (Figure 6(a)). But preincubation with C-1 did not inhibit the beneficial effects of Rb1 on PKC expression (Figure 6(b)). Moreover, the expressions of caspases 3 and 9 increased significantly in ISO-treated cells, compared to control (*P* < 0.01). Rb1 effectively reduced the expressions of caspases 3 and 9 induced by ISO, compared to ISO-treated cells (*P* < 0.01). But no change was seen for caspase-8 (Figure 6(c)). These results suggested that Rb1 reduced ISO-induced expressions of PKA, PKC, caspase-3, and caspase-9, but not caspase-8 in H9c2 cells.

## 4. Discussion and Conclusions

The inhibition of cardiac apoptosis, which can lead to heart failure [19, 20], holds a promise as an effective therapeutic way for cardiovascular disease. There are many reasons for cardiac apoptosis, such as hypoxia, ischemia, and *β*-adrenergic receptor (*β*-AR) stimulation. The *β*-AR stimulation is a common reason to reduce cardiac cell survival [21, 22]. In fact, the concentration of norepinephrine gradually increased with the aggravation of heart failure in clinical [23], different *β*-AR antagonists have been observed in clinical to treat heart failure, such as carvedilol and metoprolol [24].

Natural products are one of the most important fields of drug discovery, such as Notoginseng and Ginseng which still have popular application in traditional Chinese medicine to cardiovascular diseases. Rb1, as a compound of Notoginseng and Ginseng, has been reported to inhibit neonatal rat cardiomyocytes apoptosis [15] and protect against ischaemia/reperfusion injury [25]. However, whether Rb1 reduces cardiomyocyte death through *β*-AR-stimulated apoptosis is not disclosed.

It has been reported that ISO-induced cell death was carried out by *β*-AR in cardiomyoblast H9c2 cells [2, 7]. In this study, the effect of Rb1 on the survival of H9c2 cells was evaluated by MTT assay and showed that Rb1 significantly reduced the ISO-induced cell death. Furthermore, the anti-apoptotic effect of Rb1 was verified by TUNEL assay in the left ventricle of rats and by Hoechst 33258 staining and Flow cytometry assays in H9c2 cells. It was suggested that Rb1 reduced ISO-induced cardiomyocytes apoptosis.

Previous reports has provided evidence that ISO stimulation have been described to induce cardiac cell apoptosis which depended on PKA pathway or PKC pathway [7, 18, 19, 26]. Rb1 protective effect was also known to be involved in cAMP/PKA [27, 28]. Our study demonstrated that Rb1 increased cell survival and reduced cell apoptosis stimulated with ISO, which can be inhibited partly by H89, a PKA inhibitor, but not by C-1, a PKC inhibitor. H89 increased cell death and induced apoptosis in Figures 2 and 4, respectively. However, the cotreatment of ISO+Rb1+H89 inhibited cell growth more significantly than H89 alone. This increased cells death by cotreatment may not be explained by the cytotoxicity of H89 alone. The induced cells inhibition by cotreatment of ISO+Rb1+H89 may be involved in PKA pathway. In addition, in-cell western blot assay showed that Rb1, in presence of ISO, decreased intracellular PKA and PKC expressions in H9c2 cells. Moreover, H89 decreased the beneficial effect of Rb1 on PKA expression, but C-1 did not inhibit this effect of Rb1 on PKC expression. Obviously, these findings indicated that Rb1 inhibited myocyte apoptosis induced by ISO, through PKA signaling, but not PKC signaling pathway. 

Cysteine-dependent aspartate-specific proteases (caspase) have been demonstrated to be crucial mediators in apoptotic pathway [29]. There are two well-characterized mammalian caspase activation pathways [30, 31], including the death receptor pathway (extrinsic pathway) and the mitochondria/cytochrome c-mediated pathway (intrinsic pathway); in the death receptor pathway, the death signal proteins activated the initiator caspase, caspase-8, which in turn activated downstream effector caspases such as caspase-3. In the mitochondria-mediated pathway, caspase-9 can be activated, which activated the central executioner, caspase-3. 

It has been also reported that caspase family proteases played an essential role in ISO-induced apoptosis [32, 33]. Concerning Rb1 infusion with experimental cerebral ischemia/reperfusion, caspase-3 was significantly reduced compared to ischemia rats [33]. In this study, we have shown that caspases 3 and 9 proteins were upregulated along with the occurrence of apoptosis in cardiomyocytes by treatment with ISO. This upregulation was effectively abrogated by the cotreatment with Rb1. However, there was no different expression for caspase-8. It was suggested that Rb1-reduced apoptosis was likely mediated by caspase-9 pathways, rather than caspase-8 pathways. 

In conclusion, the present study showed that Rb1 inhibited H9c2 cardiac cells against ISO-induced apoptosis. Rb1 survival effects involved PKA signaling pathway and caspase-9 pathways. In addition to its effects *in vitro*, Rb1 reduced rat heart apoptosis cells number subjected to ISO injury.

## References

[1] Geng YJ, Ishikawa Y, Vatner DE (1999). Apoptosis of cardiac myocytes in Gs*α* transgenic mice. *Circulation Research*.

[2] Krishnamurthy P, Subramanian V, Singh M, Singh K (2007). *β*1 integrins modulate *β*-adrenergic receptor-stimulated cardiac myocyte apoptosis and myocardial remodeling. *Hypertension*.

[3] Jin YT, Hasebe N, Matsusaka T (2007). Magnesium attenuates isoproterenol-induced acute cardiac dysfunction and *β*-adrenergic desensitization. *American Journal of Physiology—Heart and Circulatory Physiology*.

[4] Fan GC, Yuan Q, Song G (2006). Small heat-shock protein Hsp20 attenuates *β*-agonist-mediated cardiac remodeling through apoptosis signal-regulating kinase 1. *Circulation Research*.

[5] Oudit GY, Crackower MA, Eriksson U (2003). Phosphoinositide 3-Kinase *γ*-Deficient Mice Are Protected From Isoproterenol-Induced Heart Failure. *Circulation*.

[6] Zhou B, Wu LJ, Tashiro SI, Onodera S, Uchiumi F, Ikejima T (2006). Silibinin protects rat cardiac myocyte from isoproterenol-induced DNA damage independent on regulation of cell cycle. *Biological and Pharmaceutical Bulletin*.

[7] Zhou B, Wu LJ, Tashiro SI, Onodera S, Uchiumi F, Ikejima T (2007). Activation of extracellular signal-regulated kinase during silibinin-protected, isoproterenol-induced apoptosis in rat cardiac myocytes is tyrosine kinase pathway-mediated and protein kinase C-dependent. *Acta Pharmacologica Sinica*.

[8] Zhao XY, Li GY, Liu Y (2008). Resveratrol protects against arsenic trioxide-induced cardiotoxicity in vitro and in vivo. *British Journal of Pharmacology*.

[9] Zhang YG, Zhang HG, Zhang GY (2008). Panax notoginseng saponins attenuate atherosclerosis in rats by regulating the blood lipid profile and an anti-inflammatory action. *Clinical and Experimental Pharmacology and Physiology*.

[10] Zhao HP, Dan L, Zhang W (2010). Ginsenoside-Rb1 attenuates dilated cardiomyopathy in cTnT^R141W^ Transgenic mouse. *Journal of Pharmacological Sciences*.

[11] Park S, Ahn IS, Kwon DY, Ko BS, Jun WK (2008). Ginsenosides Rb1 and Rg1 suppress triglyceride accumulation in 3T3-L1 adipocytes and enhance *β*-cell insulin secretion and viability in min6 cells via PKA-dependent pathways. *Bioscience, Biotechnology and Biochemistry*.

[12] El-Naggar S, Liu Y, Dean DC (2009). Mutation of the Rb1 pathway leads to overexpression of mTor, constitutive phosphorylation of Akt on serine 473, resistance to anoikis, and a block in c-Raf activation. *Molecular and Cellular Biology*.

[13] Jiang QS, Huang XN, Yang GZ, Jiang XY, Zhou QX (2007). Inhibitory effect of ginsenoside Rb1 on calcineurin signal pathway in cardiomyocyte hypertrophy induced by prostaglandin F2*α*. *Acta Pharmacologica Sinica*.

[14] Kong HL, Li ZQ, Zhao YJ (2010). Ginsenoside Rb1 protects cardiomyocytes against CoCl2-induced apoptosis in neonatal rats by inhibiting mitochondria permeability transition pore opening. *Acta Pharmacologica Sinica*.

[15] Hu A, Jiao X, Gao E (2006). Chronic *β*-adrenergic receptor stimulation induces cardiac apoptosis and aggravates myocardial ischemia/reperfusion injury by provoking inducible nitric-oxide synthase-mediated nitrative stress. *Journal of Pharmacology and Experimental Therapeutics*.

[16] Zhou WH, Du MR, Dong L (2007). Cyclosporin A increases expression of matrix metalloproteinase 9 and 2 and invasiveness in vitro of the first-trimester human trophoblast cells via the mitogen-activated protein kinase pathway. *Human Reproduction*.

[17] Ding B, Abe JI, Wei H (2005). A positive feedback loop of phosphodiesterase 3 (PDE3) and inducible cAMP early repressor (ICER) leads to cardiomyocyte apoptosis. *Proceedings of the National Academy of Sciences of the United States of America*.

[18] Yan C, Ding B, Shishido T (2007). Activation of extracellular signal-regulated kinase 5 reduces cardiac apoptosis and dysfunction via inhibition of a phosphodiesterase 3A/inducible cAMP early repressor feedback loop. *Circulation Research*.

[19] Sharov VG, Sabbah HN, Shimoyama H, Goussev AV, Lesch M, Goldstein S (1996). Evidence of cardiocyte apoptosis in myocardium of dogs with chronic heart failure. *American Journal of Pathology*.

[20] Narula J, Haider N, Virmani R (1996). Apoptosis in myocytes in end-stage heart failure. *New England Journal of Medicine*.

[21] Granata R, Trovato L, Gallo MP (2009). Growth hormone-releasing hormone promotes survival of cardiac myocytes in vitro and protects against ischaemia-reperfusion injury in rat heart. *Cardiovascular Research*.

[22] Zaugg M, Xu W, Lucchinetti E, Shafiq SA, Jamali NZ, Siddiqui MAQ (2000). *β*-Adrenergic receptor subtypes differentially affect apoptosis in adult rat ventricular myocytes. *Circulation*.

[23] Cohn JN, Levine TB, Olivari MT (1984). Plasma norepinephrine as a guide to prognosis in patients with chronic congestive heart failure. *New England Journal of Medicine*.

[24] Cruickshank JM (2010). Beta-blockers and heart failure. *Indian heart journal*.

[25] Pasupathy S, Homer-Vanniasinkam S (2005). Ischaemic preconditioning protects against Ischaemia/Reperfusion injury: emerging concepts. *European Journal of Vascular and Endovascular Surgery*.

[26] Tomita H, Nazmy M, Kajimoto K, Yehia G, Molina CA, Sadoshima J (2003). Inducible cAMP early repressor (ICER) is a negative-feedback regulator of cardiac hypertrophy and an important mediator of cardiac myocyte apoptosis in response to *β*-adrenergic receptor stimulation. *Circulation Research*.

[27] Liang W, Ge S, Yang L (2010). Ginsenosides Rb1 and Rg1 promote proliferation and expression of neurotrophic factors in primary Schwann cell cultures. *Brain Research*.

[28] Xue JF, Liu ZJ, Hu JF, Chen H, Zhang JT, Chen NH (2006). Ginsenoside Rb1 promotes neurotransmitter release by modulating phosphorylation of synapsins through a cAMP-dependent protein kinase pathway. *Brain Research*.

[29] Lu D, Lian H, Zhang X (2010). LMNA E82K mutation activates FAS and mitochondrial pathways of apoptosis in heart tissue specific transgenic mice. *PLoS ONE*.

[30] Lv BF, Yu CF, Chen YY (2006). Protein tyrosine phosphatase interacting protein 51 (PTPIP51) is a novel mitochondria protein with an N-terminal mitochondrial targeting sequence and induces apoptosis. *Apoptosis*.

[31] Saji K, Fukumoto Y, Suzuki J, Fukui S, Nawata J, Shimokawa H (2007). Colchicine, a microtubule depolymerizing agent, inhibits myocardial apoptosis in rats. *Tohoku Journal of Experimental Medicine*.

[32] Tan WQ, Wang JX, Lin ZQ, Li YR, Lin Y, Li PF (2008). Novel cardiac apoptotic pathway the dephosphorylation of apoptosis repressor with caspase recruitment domain by calcineurin. *Circulation*.

[33] Orlov SN, Thorin-Trescases N, Dulin NO (1999). Activation of cAMP signaling transiently inhibits apoptosis in vascular smooth muscle cells in a site upstream of caspase-3. *Cell Death and Differentiation*.

